# Heart failure quantified by underlying cause and multiple cause of death in Brazil between 2006 and 2016

**DOI:** 10.1186/s12889-021-12173-x

**Published:** 2021-11-15

**Authors:** Paolo Blanco Villela, Sonia Carvalho Santos, Glaucia Maria Moraes de Oliveira

**Affiliations:** Department of Cardiology, Federal University of Rio de Janeiro, Rua Rodolpho Paulo Rocco 255 / 8o. Andar, Ilha do Fundão, Rio de Janeiro, RJ 21941-913 Brazil

**Keywords:** Cardiovascular disease, Heart failure, Mortality, Causality, Underlying cause of death

## Abstract

**Background:**

The Global Burden of Disease (GBD) does not produce estimates of heart failure (HF) since this condition is considered the common end to several diseases (i.e., garbage code). This study aims to analyze the interactions between underlying and multiple causes of death related to HF in Brazil and its geographic regions, by sex, from 2006 to 2016.

**Methods:**

Descriptive study of a historical series of death certificates (DCs) related to deaths that occurred in Brazil between 2006 and 2016, including both sexes and all age groups. To identify HF as the underlying cause of death or as a multiple cause of death, we considered the International Classification of Diseases (ICD) code I50 followed by any digit. We evaluated the deaths and constructed graphs by geographic region to compare with national data.

**Results:**

We included 1,074,038 DCs issued between 2006 and 2016 that included code I50 in Parts I or II of the certificate. The frequency of HF as the multiple cause of death in both sexes was nearly three times higher than the frequency of HF as an underlying cause of death; this observation remained consistent over the years. The Southeast region had the highest number of deaths in all years (about 40,000 records) and approximately double the number in the Northeast region and more than four times the number in the North region. Codes of diseases clinically unrelated to HF, such as diabetes mellitus, chronic obstructive pulmonary disease, and stroke, were mentioned in 3.11, 2.62, and 1.49% of the DCs, respectively.

**Conclusions:**

When we consider HF as the underlying cause of death, we observed an important underestimation of its impact on mortality, since when analyzed as a multiple cause of death, HF is present in almost three times more deaths recorded in Brazil from 2006 to 2016. The mentioning of conditions with little association with HF at the time of the death highlights the importance of HF as a complex syndrome with multiple components that must be considered in the analysis of mortality trends for implementation of public health management programs.

## Background

The number of individuals with cardiovascular diseases (CVDs) increased from 271 (95% uncertainty interval [UI]: 257–285) million in 1990 to 523 (95% UI 497–550) million in 2019, and the number of CVD deaths increased steadily from 12.1 (95% UI 11.4–12.6) million in 1990 to 18.6 (95% UI 17.1–19.7) million across the 21 world regions analyzed by the Global Burden of Disease (GBD) in 2019. Considering the population growth and aging, the prevalence of CVDs is estimated to increase in several geographic areas. In Brazil, the disability-adjusted life years (DALYs) lost due to ischemic heart disease (IHD), the main component of CVD mortality, ranged from 771.2 (95% UI 679.4–866.3) per 100,000 in Amazonas to 2416.2 (95% UI 2176.7–2686.2) per 100,000 in Rio de Janeiro, a 103.2% difference within the country, with important regional variations [1].

Estimates by the GBD regarding heart failure (HF) are unavailable since HF – a common end to several diseases – is frequently used as a garbage code in which death due to HF is redistributed by the conditions responsible for its occurrence. According to data from the Brazilian Mortality Information System (*Sistema de Informação sobre Mortalidade*, SIM) of the Ministry of Health, 1,185,120 deaths that occurred between 1980 and 2018 had HF listed as the underlying cause of death (49.3% [584,155] in men). Santos et at. showed that the distribution of these deaths by Brazilian geographic region showed 48,533 records in the North, 245,898 in the Northeast, 602,105 in the Southeast, 218,496 in the South, and 70,088 in the Midwest [2]. The authors highlighted that the evaluation of the underlying causes of death has an important limitation because HF-related codes are not selected in the presence of other diseases such as IHD, resulting in an underestimation of death related to HF, urging a study of HF included as multiple causes of death [2].

The aging of the population and the aggregation of multiple comorbidities to the death process require the assessment of all diseases contributing to death. Mentioning and coding the multiple conditions contributing to HF death provides an opportunity to study the deaths to which HF may have contributed and other death causes competing with HF in selecting the underlying cause. Additionally, the evaluation of multiple causes of death yields a more multidimensional, comprehensive, and up-to-date character to the study of mortality. This allows for an analysis of the relationship between the determinants of death from a complex syndrome with multiple etiologies for planning and implementation of public health policies [3, 4].

Based on these considerations, the aim of this study was to analyze the interactions between underlying and multiple causes of death related to HF according to sex in Brazil, considering the country as a whole and divided by geographic regions (federative units), from 2006 to 2016.

## Methods

This was an ecological and descriptive study of death records due to HF in Brazil between 2006 and 2016, a period of time with more mentions in the death records across all age groups and both sexes. Information on DCs across all federative units and on an annual basis was obtained from the SIM of the Ministry of Health, available on the website of the Department of Informatics of the Unified Health System (*Departamento de Informática do Sistema Único de Saúde* [DATASUS]) [5]. After downloading the database pertaining to the period between 2006 and 2016, the original files, which were in a. DBC format, were converted to a. DBF format using the software TabWin (DATASUS, Brazil). Subsequently, the files were converted to the. XLSX format using the software Calc (LibreOffice, The Document Foundation) and analyzed using Stata 13.1 (StataCorp LP, College Station, TX, USA).

Information about the population residing at each region was also obtained from the DATASUS website, [6] which offers censual data from the Brazilian Institute of Geography and Statistics (*Instituto Brasileiro de Geografia e Estatística*, IBGE) from 2000 and 2010, intercensal projections until 2012, and population projections from 2013 onwards.

We identified the occurrence of HF listed in the DC as the presence of code I50 (followed by any digit) of the 10th revision of the International Statistical Classification of Diseases and Related Health Problems (ICD-10) listed in Parts I or II of the certificate. We excluded those in which the field for information regarding the sex of the decedent was either empty or marked as “ignored.” Since each line in Parts I (Lines A, B, C, and D) and II of the DC may display up to four different ICD-10 codes, we subdivided these lines into four subparts, each with a single code, so that the frequency of each code could be assessed independently.

We analyzed the absolute numbers of DCs at a national level, and the absolute numbers of DCs and mortality rates per 100,000 inhabitants in each geographic region (identified by federative units), according to the selection of HF as the underlying cause or it’s mention in any part of DC - as a multiple cause of death. When they are mentioned in more than one line in DC, it was considered just once.

All codes cited in Parts I and II of the selected DCs were evaluated and we also performed this analysis by geographic region and built separate graphs for comparison of national data. The codes I50.0, I50.1, and I50.9 were analyzed together and represented by the code I50x.

The data were analyzed using Stata (StataCorp LP), and the graphs and tables were built using Excel (Office 365, Microsoft, Seattle, WA, USA).

## Results

Between 2006 and 2016, we identified 1,074,038 DCs with code I50x cited in Parts I and II. Of these, 120 had improper information in the field “sex,” and were not included when analysis by sex were performed.

**Fig. 1:**
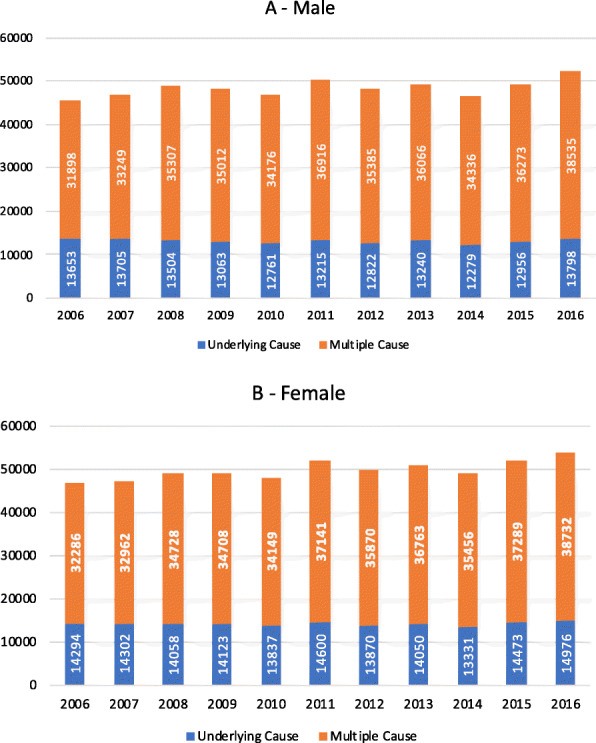
Number of death certificates mentions heart failure (I50) as multiple causes of death and the underlying cause of death. **A** male sex; **B** female sex. Brazil, 2006–2016. Orange bars includes mentions in all lines of death certificate and did not include the selected underlying cause of death after application of selection rules, which are represented by blue bars

As shown in Fig. 1, the frequency of HF as a multiple cause of death in both sexes was nearly three times higher than the frequency of HF as the underlying cause of death (Fig. 1A and B). This observation remained constant across the years. Both scenarios (underlying and multiple cause) show a similar temporal trend in both sexes, with a greater number of DCs from women (Fig. 1A and B). In both scenarios, the curves are similar along the 11 years of observation and increased over the last 3 years (Fig. 1A and B). For example: in Fig. 1, this ratio is 2.34 in men, in 2006 (absolute number 13,653 as underlying cause and absolute number 31,898 as multiple causes of death).

**Fig. 2:**
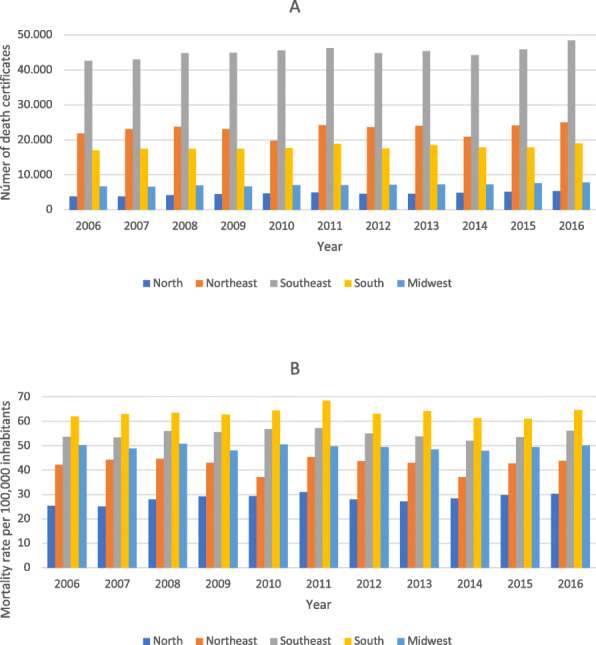
**A** Number of death certificates with heart failure cited as a multiple cause of death by region; **B** Mortality rate with heart failure cited as a multiple case of death per 100,000 inhabitants according to geographic region. Brazil, 2006–2016

Quantifying the number of DCs listing HF as a multiple cause of death in each geographic region and across all regions showed little variability over the years (Fig. 2A). In all years, the Southeast region had the highest number of DCs (about 40,000 records each year), which was approximately twice the number of DCs in the Northeast region and more than four times that in the North region. In all regions, the number of DCs increased over the last 3 years of observation (Fig. 2A). Figure 2B shows an overall balance in mortality rates with HF as a multiple cause of death per 100,000 inhabitants across geographic regions. Notably, the South, Southeast, and Midwest regions had the highest mortality rates in all years (Fig. 2B), while the regions showed overall less temporal fluctuations when compared with their own absolute values.

**Fig. 3:**
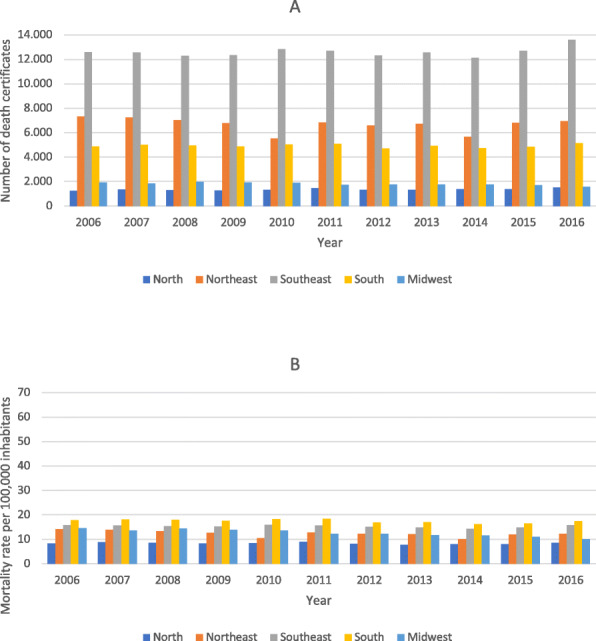
**A** Number of death certificates with heart failure cited as the underlying cause of death, according to geographic region; **B** mortality rate of heart failure cited as the underlying cause of death per 100,000 inhabitants according to geographic region. Brazil, 2006–2016

Figure 3 shows HF as the underlying cause of death, considering the absolute numbers of DCs and the mortality rate per 100,000 inhabitants in each region. Among DCs with HF cited as the underlying cause of death, the number of DCs by geographic region and mortality rates per 100,000 inhabitants showed lower rates compared with the corresponding DCs in which HF was cited as a multiple cause of death, although the trends were overall similar along the analyzed period, with emphasis on the South region, which had the highest mortality rate (Fig. 3B).

Of the 296,715 DCs with HF (ICD Codes I50.0, I50.1. I50,9) cited as the underlying cause of death, 886,442 mentions were found, and the two codes most frequently cited in Parts I and II were directly related to HF (I50.0 and I50.9), listed in 33.5% of the deaths. Table 1 describes the associated causes of death related to heart failure as the underlying cause. Of note, codes of unspecified diseases such as “pulmonary edema” and “cardiogenic shock” were listed in about 5% of the DCs, while “essential hypertension” – a clinical condition frequently associated with HF – was only cited in 3.15% of the DCs (Table 1). Other codes cited in less than 2.75% of the DCs were combined into the category “others” in Table 1.

**Table 1:** Frequency of codes listed in Parts I and II of death certificates with heart failure (ICD I50) listed as the underlying cause of death (Brazil, 2006–2016)

ICD	Description	n	(%)
I50.0	Heart failure	158,470	17.9
I50.9	Heart failure, unspecified	137,912	15.6
J81X	Pulmonary edema, not elsewhere classified	47,607	5.4
R57.0	Cardiogenic shock	46,734	5.3
R09.2	Respiratory arrest	46,520	5.3
J96.0	Acute respiratory failure	36,986	4.2
J96.9	Respiratory failure, unspecified	34,099	3.8
J18.9	Pneumonia, unspecified organism	28,018	3.2
I10X	Essential (primary) hypertension	27,903	3.1
A41.9	Sepsis, unspecified organism	24,350	2.7
	Others	297,443	66.4
	Total	886,042	100.00

Overall, HF was listed in any line of Parts I and II 1,074,038 times in all DCs, and the codes most frequently mentioned in these cases were those with the term “heart failure” (I50.0, I11.0, and I50.9) (Table 2). Of note, the presence of codes related to diseases without a close clinical relationship with HF, such as diabetes mellitus, chronic obstructive pulmonary disease (COPD), and stroke, cited in 3.11, 2.62, and 1.49% of the DCs, respectively (Table 2). Other codes cited in less than 0.89% of the DCs were combined into the category “other codes not mentioned above” in Table 2.

**Table 2:** Frequency of codes listed as the underlying cause of death in death certificates with heart failure (ICD I50) present anywhere in Parts I and II (Brazil, 2006–2016)

ICD	Description	n	(%)
I50.0	Heart failure	157,769	14.7
I11.0	Hypertensive heart disease with (congestive) heart failure	121,289	11.3
I50.9	Heart failure, unspecified	118,261	11.0
I21.9	Acute myocardial infarction, unspecified	90,422	8.4
E14.9	Unspecified diabetes mellitus - without complications	33,392	3.1
J18.9	Pneumonia, unspecified organism	32,913	3.1
I42.0	Dilated cardiomyopathy	31,053	2.9
J44.9	Chronic obstructive pulmonary disease, unspecified	28,151	2.6
I50.1	Left ventricular failure	20,685	1.9
B57.2	Chagas disease (chronic) with heart involvement	18,366	1.7
I64	Stroke, not specified as hemorrhage or infarction	16,011	1.5
J44.0	Chronic obstructive pulmonary disease with acute lower respiratory infection	15,791	1.5
I251	Atherosclerotic heart disease	15,426	1.4
I10	Essential (primary) hypertension	12,430	1.2
J18.0	Pneumonia, organism unspecified	11,590	1.1
I13.2	Hypertensive heart and renal disease with both (congestive) heart failure and renal failure	11,269	1.0
I25.9	Chronic ischemic heart disease, unspecified	10,651	1.0
I25.5	Ischemic cardiomyopathy	10,360	1.0
E14.2	Unspecified diabetes mellitus - with renal complications	9616	0.9
I24.8	Other forms of acute ischemic heart disease	9523	0.9
(…)	Other codes not mentioned above	299,070	27.9
	Total	1,074,038	100.00

**Fig. 4:**
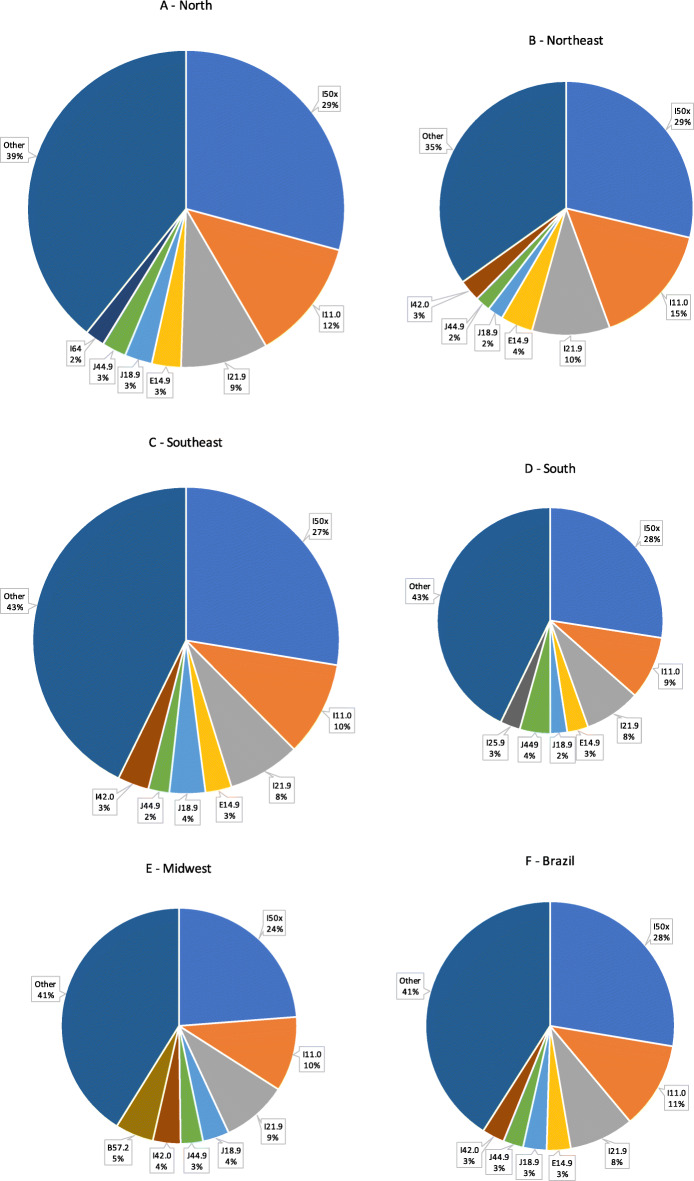
Frequency of ICD-10 codes cited as the underlying cause of death when code I50 was mentioned anywhere in Parts I and II of the death certificate, according to geographic region (**A**-**E**) and in the entire country (**F**), Brazil, 2006–2016. Total number of deaths: North: 50,555; Northeast: 253,182; Southeast: 495,976; South: 196,172; Midwest: 78,153; Brazil: 1,074,038. ICD-10 codes: I50.x: congestive heart failure; I11.0: hypertensive heart disease with (congestive) heart failure; I21.9: acute myocardial infarction, unspecified; E14.9: unspecified diabetes mellitus; J18.9: pneumonia, unspecified; J44.9: chronic obstructive pulmonary disease, unspecified; I64: stroke, not specified as hemorrhage or infarction; I42.0: dilated cardiomyopathy; I25.9: chronic ischemic heart disease, unspecified; B57.2: Chagas disease (chronic) with heart involvement

Fig. 4 displays, by geographic region, the codes most frequently listed as the underlying cause of death in all DCs that listed the code I50 in Parts I or II. Despite the evident predominance of the I50x code in all regions, there was an increased frequency of codes I11.0 and I21.9, related to hypertensive and ischemic diseases, respectively, mainly in the North (Fig. 4A) and Northeast (Fig. 4B) regions of the country. The Southeast (Fig. 4C), South (Fig. 4D), and Midwest (Fig. 4E) regions showed similar patterns to that observed at a national level (Fig. 4F).

## Discussion

In some regions across the world, the age-adjusted incidence of HF has stabilized and appears to be decreasing, but the total number of patients living with HF is increasing, reflecting the chronic course of the disease associated with population aging and growth. Data about the epidemiology of HF are limited, especially in middle-income countries like Brazil, where the prevalence of HF is considered to be growing, and an increasing association of HF with conditions such as rheumatic disease, Chagas disease, hypertension, and others has been reported [7]. A study evaluating the mortality rates with HF listed as the underlying cause of death in Brazil has shown a progressive decline from 2008 to 2018 and similar levels in 2018 across all geographic regions and federative units, along with higher rates in men throughout the period [2]. When we quantified HF as the underlying cause of death in Brazil from 2006 to 2016, we observed an important underestimation of the impact of this condition on mortality, since quantify of HF as a multiple cause of death found almost three times more DCs.

Another Brazilian study analyzing deaths from HF between 2008 and 2012 compared two models redistributing HF deaths to specific causes of death in the age group of 55 years or more. One model, in which the deaths were redistributed based on data from hospital records, redistributed 45.8% of the deaths to HF based on the principal diagnosis at the hospital. The other model (method of multiple causes of death) redistributed the underlying cause of death in records in which HF was cited as the underlying cause of death to hypertensive heart and kidney diseases (25.3%), IHD (22.6%), and diabetes (9.6%) [4].

In our population-based study in adults of both sexes, we also observed that HF was the most frequent diagnosis (33.5% of the cases) when this condition was listed as the underlying cause of death (Table 1). On the other hand, the frequency of HF cited as the underlying cause of death when HF was present anywhere in Parts I or II of the DC decreased to 25.7%, followed by hypertensive heart disease with HF, IHD, and diabetes (Table 2). These findings highlight the contribution of HF as a complex syndrome with multiple components that must be considered in analyses of mortality trends performed with the objective of implementing public health management programs.

Of note, although Chagas’ disease with cardiac involvement is reported to be highly prevalent in Brazil and Latin America, [8, 9] this condition was infrequently listed as a multiple cause of death (1.7%) (Table 2), emphasizing the concept that death is multifactorial in patients with HF.

Considering the total number of DCs with HF as the underlying cause of death or as a multiple cause of death, women predominated throughout the period (Fig. 1), particularly when HF was listed as the underlying cause of death. A study inferring the underlying cause of death in DCs citing HF as the underlying cause of death in the age group of 55 years and more in Maryland, Minnesota, Mississippi, and North Carolina, also found that women predominated among the decedents (53.3%). The deaths were redistributed to IHD in 37.1% of the cases, to other CVDs in 10.8%, COPD in 8%, cancer in 6.7%, diabetes in 4.9%, hypertensive kidney and heart disease in 4.5%, and cerebrovascular disease in 4.4% of them [10]. The authors observed differences across states and ethnic groups. When we analyzed the Brazilian data of Bierrenbach et al.*, we realized* that there was a need to evaluate the frequency of HF listed as a multiple cause of death in different regions of the country.

A study using coarsened exact matching to redistribute deaths records certified to HF (ICD-10, I50) in the US, Mexico, and Brazil (age group 20–84 years, both sexes) assigned most deaths to IHD (53, 26, and 22%, respectively), followed by hypertensive heart disease (16, 23, and 7%, respectively), and diabetes (13, 9, and 6%, respectively) [11]. Another study proposing a method to reclassify garbage-code deaths into causes more meaningful for public health application using a US database redistributed 48% of the HF deaths to other cardiovascular diseases, 25% to IHD, and 15% to chronic respiratory diseases. The authors also observed that the multiple causes associated with HF varied according to sex and age group [12].

In the present study, the number of DCs with HF listed as a multiple cause of death was higher in the Southeast region across the entire period, especially in 2016. In contrast, the mortality rate of HF as a multiple cause of death (per 100,000 inhabitants) was higher during the entire period in the South region, with a peak in the year 2011, probably due to the aging of the population and the quality of the information cited in DCs in the South. A study analyzing the observed and predicted standardized mortality related to HF as an underlying cause from 2001 to 2030 in Brazil estimated a progressive reduction in the last years to about 6.1 and 6.2% in men and women, respectively, although the authors stressed that the absolute number of deaths from HF should increase over time due to population growth and aging. They observed higher rates in the South and Southeast regions, which they attributed to greater access to the health care system by patients with chronic diseases and to increased life expectancy in these regions [13].

## Conclusions

The evaluation of HF as a multiple cause of death is fundamental since the official data considering only the underlying cause of death substantially underestimates the impact of this condition on mortality data. The report in the DC of conditions at the time of death that have little association with HF, as found in the present study, highlights the importance of HF as a complex syndrome with multiple components that need to be considered in the analysis of mortality trends for implementation of public health management programs.

## Data Availability

All the data sets generated and/or analyzed during the present study were obtained from the Mortality Information System of the Brazilian Ministry of Health and available on the DATASUS website: http://www2.datasus.gov.br/DATASUS/index.php.

## Abbreviations

COPD: Chronic obstructive pulmonary disease
CVDs: Cardiovascular diseases
DALY: Disability-adjusted life years
DC: Death certificates
GBD: Global Burden of Disease
HF: Heart Failure
ICD-10: 10th Revision of the International Statistical Classification of Diseases
IHD: Ischemic Heart Disease
UI: Uncertainty interval
